# Transplacental Transmission of Cytomegalovirus (CMV) in Pregnant Women with Positive Anti-CMV IgG and Negative Anti-CMV IgM in Highly CMV Seropositive Region

**DOI:** 10.3390/pathogens14090894

**Published:** 2025-09-05

**Authors:** Jie Tang, Hongxia Wei, Yimin Dai, Yuqian Luo, Yali Hu, Yi-Hua Zhou, Nacheng Lin, Aimin Liu

**Affiliations:** 1 Department of Obstetrics and Gynecology, Wujin Hospital Affiliated with Jiangsu University, Changzhou 213017, China; xiaoyao1979@sina.com; 2 Department of Obstetrics and Gynecology, The Wujin Clinical College of Xuzhou Medical University, Changzhou 213017, China; 3 Department of Laboratory Medicine, Nanjing Drum Tower Hospital, Nanjing University Medical School, Nanjing 210008, China; xiaoxia66260@163.com; 4 Department of Obstetrics and Gynecology, Nanjing Drum Tower Hospital, Nanjing University Medical School, Nanjing 210008, China; nj_daiyimin@126.com (Y.D.); dtylhu@126.com (Y.H.); 5 Department of Core Laboratory, Nanjing Drum Tower Hospital, Nanjing University Medical School, Nanjing 210008, China; yuqianluo31@foxmail.com (Y.L.); zgr03summer@126.com (Y.-H.Z.); 6 Department of Infectious Diseases, Nanjing Drum Tower Hospital, Nanjing University Medical School, Nanjing 210008, China

**Keywords:** cytomegalovirus, non-primary infection, transplacental transmission, newborn, long-term sequelae

## Abstract

Primary or recurrent infection of cytomegalovirus (CMV) in pregnant women may cause transplacental transmission to fetuses. We aimed to investigate the rate of transplacental CMV transmission in women with positive anti-CMV IgG and negative anti-CMV IgM and its impact on newborns. Pregnant women with positive anti-CMV IgG and negative anti-CMV IgM during the first or second trimester who delivered by Cesarean section were included. Amniotic fluid collected during the Cesarean section was tested for CMV DNA with quantitative real-time polymerase chain reaction. CMV IgG and IgM were measured with enzyme-linked immunosorbent assay. A total of 695 pregnant women were enrolled between April 2019 and February 2023. Of them, 567 (81.6%) were single pregnancies and 128 (18.4%) were twin pregnancies, and 594 (85.5%) were full-term pregnancies and 101 (14.5%) were premature pregnancies. Of the 823 newborns, 7 (0.9%) were CMV DNA positive in amniotic fluid, demonstrating the transplacental CMV transmission. One of these seven neonates was diagnosed with intrauterine growth restriction at gestation week 25^+1^ and at birth at a gestational age of 30^+2^ weeks. However, all seven children had normal hearing, vision, and neurodevelopment at the age of 18–56 months. Transplacental CMV transmission may occur in offspring of pregnant women with positive anti-CMV IgG and negative anti-CMV IgM, but the long-term sequelae appear to be minimal.

## 1. Introduction

Maternal infection during pregnancy may cause adverse pregnancy and neonatal outcomes [1,2]. Infection of human cytomegalovirus (CMV), a member of the Herpesviridae family, is characterized by its latent nature like other herpes viruses, the absence of viral progeny, and little harmful outcome in immunocompetent individuals. However, reactivation of the preexisting virus or reinfection of a new CMV isolate may occur during pregnancy because of suppressed T cell immune functions. Transplacental transmission of maternal CMV to the fetus is a main cause of non-genetic fetal/neonatal abnormalities in industrialized countries [3]. Approximately half of women at childbearing age in these countries are seronegative for CMV and are prone to primary infection during pregnancy [4], and an estimated 9.9–15.4% of severe neonatal adverse outcomes may be associated with congenital CMV infection [5,6]. Transplacental CMV transmission is also an important cause for fetal/neonatal abnormalities in the developing world [7,8], although the proportion of CMV infection in terminated fetuses with severe malformation appeared to be relatively low [9].

Transplacental CMV transmission mainly occurs in pregnant women with active infection, which is defined by positive CMV IgG and IgM. Active CMV infection may be primary or non-primary. Transplacental CMV transmission rate is estimated at 30–40% in pregnancy with primary infection and 1–2% in pregnancy with non-primary infection [10,11,12], which includes either reactivation or reinfection. However, non-primary active infection is not necessarily characterized by the presence of CMV IgM and may consequently be positive for CMV IgG alone. Pregnant women with positive CMV IgG alone are considered less likely to have transplacental transmission. A recent study of 9661 newborns of women with preconception immunity in Italy showed that the congenital CMV infection rate was as low as 0.19% [13]. However, the data on transplacental CMV transmission in pregnant women with positive CMV IgG alone in high seroprevalence regions are scant, and the long-term influence of the transplacental transmission on the outcomes of fetuses/neonates under such circumstances is less studied. In the present study, we investigated the transplacental CMV transmission in pregnant women with positive CMV IgG alone by detection of CMV DNA in amniotic fluid collected during the Cesarean section.

## 2. Materials and Methods

### 2.1. Study Population and Sample Collection

Renal epithelium is one of the main targets of CMV replication. Amniotic fluid is mainly composed of fetal urine. Virions excreted in the fetal urine may accumulate in amniotic fluid. Thus, detection of CMV DNA in amniotic fluid by quantitative real-time polymerase chain reaction (qPCR) is considered highly sensitive to define transplacental CMV transmission. Amniotic fluid collected during spontaneous delivery is inevitably contaminated by vaginal secretion, which may contain CMV [14,15]. Therefore, in the present study, we collected amniotic fluid during Cesarean section to detect CMV DNA so that the contamination by vaginal secretion may be avoided.

This was a prospective cohort study conducted at two tertiary hospitals in Jiangsu province, China. Based on the incidence of 1–2% transplacental CMV transmission [16], a range of from 409 to 497 participants would be required with the maximum allowable error at 1–1.5%. Pregnant women who were CMV IgG positive and CMV IgM negative and tested during the first (the first prenatal care during gestation, 12 weeks) or second (gestation 13–27 weeks) trimester and who underwent Cesarean section were eligible for this study. Those who were HIV positive or received immunosuppression therapy were excluded.

Peripheral blood samples (~5 mL) of the pregnant women were collected on the day of (elective Cesarean section) or just (emergency Cesarean section) before the surgery. Amniotic fluid samples (~10 mL) were collected with a syringe with or without a needle, depending upon whether the amniotic cavity was opened or not during the Cesarean section, with caution not to injure the fetus. Cord blood samples (~5 mL) were collected from the placental umbilical cord vessel immediately after the placenta was delivered. To ensure as many amniotic fluid and cord blood samples as possible were collected, an additional nurse was arranged to collect the samples during each Cesarean section. The blood samples were separated into serum and coagulated part by centrifugation at 4 °C, and the serum samples were aliquoted in 1.5 mL tubes and kept at −30 °C. Amniotic fluid samples were directly aliquoted in 1.5 mL tubes and stored at −30 °C.

This study was approved by the Institutional Review Board of the Ethics Committee of Wujin Hospital Affiliated with Jiangsu University on 3 February 2021, and the ethical approval of Nanjing Drum Tower Hospital was applied later and approved on 13 August 2024. However, we performed this study in accordance with the ethical standards of the 1964 Declaration of Helsinki and its later amendments. Since we just tested CMV IgG and IgM and CMV DNA in the participants and did not take any other intervention during pregnancy, and Cesarean section was determined based on medical indications, but not for this study, all pregnancies went to term without harm to both pregnant women and their offspring. In addition, the cost for testing anti-CMV IgG and IgM and CMV DNA was covered by fundings, but not by the pregnant women. Written informed consent was obtained from each pregnant woman during hospitalization before the Cesarean section.

### 2.2. Serological Tests of CMV IgG and IgM

Serum samples were tested for CMV IgG and IgM with enzyme-linked immunosorbent assay kits (Bell Biological Technology, Beijing, China) as described previously [15]. The performance (specificity and sensitivity) of the kits is comparable to that of the kits manufactured by Dia.Pro Diagnostic Bioprobes (Milano, Italy) that were used in our previous investigation [8,9,17]. The microplate in the CMV IgG kit was coated with purified inactivated CMV, and CMV IgG was detected with horseradish-conjugated mouse anti-human IgG. The microplate in the CMV IgM kit was coated with mouse anti-human IgM (μ chain), and CMV IgM was detected by horseradish-conjugated recombinant CMV p52 and p65 proteins. Each serum was diluted 1:11. Based on the manufacturer’s instructions, the cut-off value for CMV IgG and IgM was equal to or higher than the sum of 0.1 and the average values of optical density at a wavelength of 450 nm (OD450) of three negative controls in each independent assay.

CMV IgG avidity index (AI) was tested by urea denaturation with the CMV IgG kit (Bell Biological Technology) as described elsewhere [8,9,18,19]. IgG AI < 30% was considered as low, 30–50% as intermediate, and >50% as high.

### 2.3. Detection of CMV DNA

CMV DNA in amniotic fluid was measured by a commercially available CMV qPCR diagnostic kit (Aikang, Hangzhou, China) on an ABI StepOne Plus Sequence Detection System (Applied Biosystems, Foster City, CA, USA) as described previously [9,20]. Briefly, one milliliter of amniotic fluid was centrifuged at 15,000× *g* at 4 °C for 15 min, and 0.1 mL of pellet suspension was used to extract total DNA by the extraction agents in the kit. For detection of CMV DNA in the blood samples, 0.1 mL serum was used to extract total DNA. The positive control, negative control, and six calibrations (2–7 log copies/mL) were included in each test.

### 2.4. Statistical Analysis

Continuous data were presented as mean ± standard deviation (SD). Categorical variables were reported as number and percentage and compared by the χ^2^ test or Fisher’s exact test where appropriate. A two-sided *p*-value of <0.05 was considered significant. All statistical analyses were conducted using SPSS 25.0 (Chicago, IL, USA).

## 3. Results

### 3.1. General Characteristics of Participants

**Figure 1:**
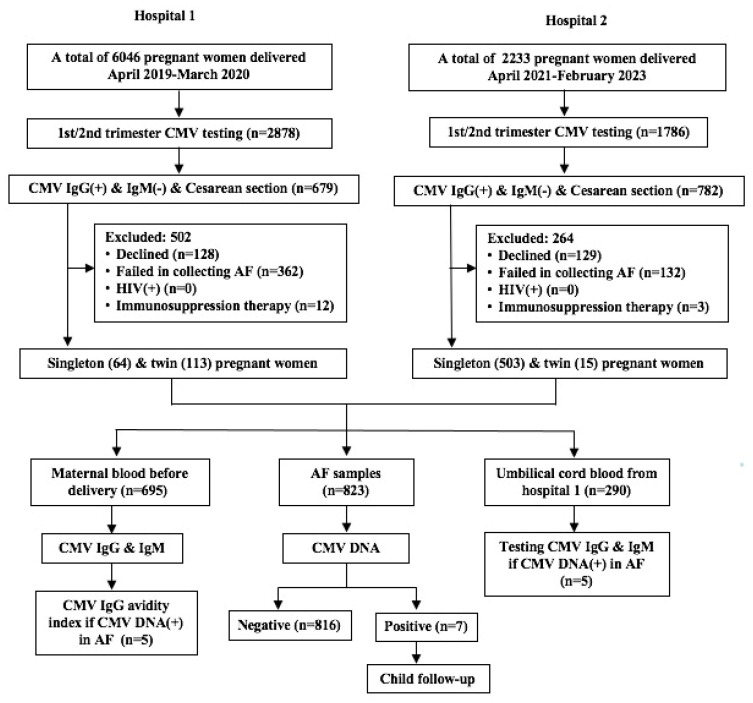
Flow chart of subject enrollment. AF, amniotic fluid.

During the period of 1 April 2019 to 31 March 2020 in a hospital, a total of 679 pregnant women who were CMV IgG positive and CMV IgM negative, tested in the first or second trimester, and who received a Cesarean section were eligible for the study (Figure 1). Of them, amniotic fluid and cord blood samples were collected from each of the 64 single pregnancies and 113 twin pregnancies with live birth (Figure 1). During the period of 1 April 2021 through 28 February 2023, a total of 782 pregnant women in another hospital were eligible for the study (Figure 1). Of them, amniotic fluid samples were collected from 518 pregnant women (15 twin pregnancies), yet the cord blood samples were not collected (Figure 1). Thus, a total of 695 pregnant women were enrolled, with 567 (81.6%) single pregnancies and 128 (18.4%) twin pregnancies. Their average age was 30.6 ± 4.7 years. The most common indications for Cesarean section were scarred uterus (48.1%) and twin pregnancy (19.7%) (Table S1). Of them, 585 (84.2%) were elective Cesarean sections and 110 (15.8%) were emergency Cesarean sections, and 594 (85.5%) were full-term deliveries and 101 (14.5%) were premature deliveries. The premature delivery rate (55.5%, 76/137) of twin pregnancy was significantly higher than that (4.5%, 25/558) of single pregnancy (χ^2^ = 230.29, *p* < 0.001).

### 3.2. Positive Rate of CMV DNA in Amniotic Fluid

In total, 695 women had 823 live newborns because of the 128 women with twin pregnancies. Of the 823 amniotic fluid samples, 7 (0.9%) were CMV DNA positive (Figure 1). Among these seven newborns, three were singletons and four (two pairs) were twins. The transplacental transmission rate (1.6%, 4/256) in twin infants was slightly higher than that (0.5%, 3/567) in singleton infants, although the difference was not statistically significant (χ^2^ = 1.128, *p* = 0.288). The viral load ranged from 2.54 × 10^2^ to 5.81 × 10^7^ copies/mL (Table 1). All three single births were full term, and the four twins (two pairs) were preterm at gestational ages 30^+2^ and 36^+1^ weeks, respectively (Table 1).

In addition, all five mothers of seven newborns with positive CMV DNA in amniotic fluid had no detectable CMV DNA in their peripheral blood collected just before Cesarean section. The cord blood samples from five infants (one singleton and four twins) were also CMV DNA negative.

### 3.3. CMV IgG and IgM Status in Pregnant Women and Newborns with Positive CMV DNA

While we enrolled pregnant women with CMV IgG positive and CMV IgM negative during the first or second trimester, we further tested CMV IgG and IgM in them just before the Cesarean section. All 695 pregnant women were still CMV IgG positive, and 11 (1.6%) of them became positive for CMV IgM. The OD450 values of these 11 samples ranged from 0.1540 to 0.4106, while the cut-off value for the positive CMV IgM ranged from 0.1500 to 0.1522 in the performance of the assays. Thus, all these 11 serum samples collected just before the Cesarean section were weakly positive for CMV IgM. On the other hand, all five women with positive CMV DNA in their newborn infants’ amniotic fluid were negative for CMV IgM just before delivery.

We further measured the CMV IgG avidity index in the serum samples from the 11 women who showed CMV IgM positive just before delivery and from the 5 women whose newborns were CMV DNA positive. The CMV IgG avidity index of each serum was higher than 50%.

Moreover, the umbilical serum samples from five (one singleton and two pairs of twins) of the seven newborns with positive CMV DNA were available, and we tested the CMV IgG and IgM. While all five samples were CMV IgG positive, three (one singleton and one pair of twins) samples were also CMV IgM positive (Table 1). The OD450 values of these three samples were 0.2537 and 0.2732 (one pair of twins) and 1.3817 (the singleton), respectively. Another pair of twins were CMV IgM negative with OD450 values of 0.0545 and 0.0495, much lower than the cut-off value.

### 3.4. Antenatal and Neonatal Features and Follow-Up Outcomes of CMV-Infected Children

Among the five women with positive CMV DNA in their amniotic fluid, routine prenatal B ultrasound screening revealed intrauterine growth restriction (IUGR) in one of the twins in a pregnant woman (#1) at gestation week 25^+1^ and did not reveal obviously abnormal findings in four other pregnant women (Table 1). This woman then underwent weekly prenatal B ultrasound screening, and abnormal umbilical blood flow had been observed in this fetus with IUGR at gestation week 29^+5^. The pregnancy in this woman was terminated by elective Cesarean section at gestation week 30^+2^.

The birth weight, body length, and Apgar scores of the seven neonates with positive CMV DNA in amniotic fluid are shown in Table 1. None of the seven neonates had petechiae, tachypnea, hepatomegaly, splenomegaly, thrombocytopenia, seizures, microcephaly, or hypotonia at birth. The hearing tests within 3 days after birth were all normal. However, both twin neonates born to a woman (#1) developed neonatal pneumonia and respiratory distress syndrome. Thus, they were admitted to the neonatal intensive care unit in a referral neonate center. During hospitalization, both of them developed necrotizing enteritis, and the neonate with IUGR underwent ileum perforation. Neither of them received antiviral therapy against CMV based on the negative results of CMV DNA in their blood samples. These two infants were discharged from the hospital after comprehensive treatments for 20 and 46 days, respectively.

All seven children were followed up at least once at the age of 18–56 months (Table 1). They had normal vision, hearing, and mental development, and none of them had neurological sequelae.

## 4. Discussion

This study estimated the rate of transplacental CMV transmission in pregnant women with positive CMV IgG and negative CMV IgM by detecting CMV DNA in amniotic fluid collected during Cesarean section. We revealed that transplacental CMV transmission occurred in 0.9% (7/823) of newborns. While a pair of preterm twins had severe clinical manifestations shortly after birth, all seven children had no severe sequelae at the age of 18 to 56 months. Our results indicate that transplacental CMV transmission in pregnant women with positive CMV IgG and negative CMV IgM may have minimal severe long-term sequelae.

Transplacental CMV transmission rate varies worldwide, depending upon the different countries and different populations, ranging from less than 1% in developed countries to 1–5% in developing countries [16]. In China, the overall seroprevalence of CMV infection in pregnant women is over 98% [8,20]; thus, primary CMV infection during pregnancy is rare, and transplacental CMV transmission mostly occurs in pregnant women with recurrent CMV infection. Because of the different assays used to define the transmission, the reported rates varied considerably in China, from as low as 0.23% (4/1756) based on the detection of CMV DNA in umbilical blood samples [21] to 1.59% (107/6733) determined by CMV DNA in saliva collected within 3 days after birth [22]. Recently, Huang et al. reported that the transmission rate was 1.33% (83/6228) based on testing CMV DNA in urine and/or saliva 13 days after birth [20]; however, a considerable proportion of positive CMV DNA in saliva may be false positive because the same authors reported a much lower defined transmission rate [23]. This situation is also reported by other studies [13,24]. In other parts of the world with CMV seroprevalence > 95% among pregnant women, the transplacental transmission rate was 1.08% (87/8047) [25]. Generally, it is considered that 1–2% of pregnant women with recurrent infection may have transplacental CMV transmission [10,12]. In the present study, we revealed a transmission rate of 0.9% in pregnant women with positive CMV IgG and negative CMV IgM. Because of the high sensitivity and almost 100% specificity, detection of CMV DNA in amniotic fluids has been recognized as the gold standard for prenatal diagnosis of transplacental CMV transmission [26,27]. Thus, the 0.9% transmission rate determined by testing CMV DNA in amniotic fluids collected during Cesarean section in the present study should reliably reflect the real situation of transplacental CMV in the pregnant women with positive CMV IgG and negative CMV IgM. In addition, similar to the finding that more congenital CMV infections occurred in twin infants in Brazil [28], we found that the transmission of CMV in twin pregnancy was somewhat higher than that in singleton pregnancy, although the difference was not statistically significant.

In pregnant women with preexisting CMV IgG, transplacental CMV transmission is generally considered the consequence of recurrent CMV infection during pregnancy. Clinically, positive CMV IgM is a marker for recurrent CMV infection in pregnant women with preexisting CMV IgG. However, we found in this study that pregnant women with positive CMV IgG alone still transmitted CMV to their offspring in utero, which was also observed previously [13,20,25,29]. This indicates that reactivation or reinfection of CMV may not be positive for CMV IgM, as studies showed that CMV IgM appears to have no association with non-primary infection [30]. Nevertheless, pregnant women with preexisting CMV IgG may cause transplacental CMV transmission, regardless of positive or negative CMV IgM.

Traditionally, it is considered that transplacental CMV transmission following primary infection during pregnancy causes many more symptomatic CMV infections in newborns and severe long-term sequelae than that after recurrent infection [31]. Recently, it is considered that once transplacental CMV transmission occurs, severe long-term sequelae such as sensorineural hearing loss and neurodevelopmental impairment are similar between maternal primary and non-primary infection [32,33]. However, in our study, none of the seven children with transplacental CMV infection had long-term sequelae, indicating that previous CMV infection may provide partial protection to lower the risk of transplacental transmission and to alleviate the severity of diseases [31]. Thus, more studies in highly CMV seroprevalence populations are required to clarify whether transplacental CMV infection in pregnant women with preexisting CMV IgG causes similar severe long-term sequelae as that in pregnant women with primary CMV infection.

There are several limitations in our study. First, we did not test CMV DNA in the urine of newborns within 14 days after birth to confirm the transplacental CMV infection. However, we measured CMV IgM in five umbilical blood samples, and three of them were positive, adding more evidence of the transplacental infection. Second, the timing of blood collection may affect the results of CMV IgG and IgM. In primary infection, CMV IgM antibodies usually appear within 1–2 weeks after infection and may become undetectable after 4–12 weeks, whereas CMV IgG antibodies develop a few days after the appearance of IgM and persist for life [11]. Thus, the pregnant women who tested positive for CMV IgG and negative for CMV IgM in the second trimester had the potential to be primarily infected with CMV in the first trimester. In addition, we did not measure the CMV IgG avidity during the pregnancy to ascertain the non-primary CMV infection, since a small proportion of primary CMV may not produce detectable specific IgM, or the specific IgM may become undetectable after 4–12 weeks [18,34]. However, we considered that the pregnant women included in this study were less likely to have had primary CMV infection during pregnancy, since nearly 100% of the Chinese women of reproductive age have had a previous CMV infection [35]. Third, the timing of transplacental CMV transmission in the seven infected children was unknown.

## 5. Conclusions

We revealed that transplacental CMV transmission occurs in approximately 1% of pregnant women with positive CMV IgG and negative CMV IgM. However, the affected newborns appear to have no severe long-term neurological sequelae and have normal development. Whether transplacental CMV transmission resulting from the non-primary infection has the same severe long-term sequelae as those caused by the primary infection merits further investigation.

## Figures and Tables

**Table 1:** Characteristics of pregnant women and children with positive CMV DNA in amniotic fluid.

	Subject 1		Subject 2		Subject 3	Subject 4	Subject 5
Maternal characteristics							
Age (y)	29		28		36	29	27
Gestational week (GW)	30^+2^		36^+1^		38^+2^	37^+5^	39^+4^
Pregnancy/delivery	G2P1		G2P0		G4P3	G2P0	G1P0
Twin/singleton	Twin		Twin		Singleton	Singleton	Singleton
Reasons for Cesarean section	Abnormal umbilical blood flow in one of the twins		Scarred uterus		Scarred uterus, preeclampsia, macrosomia	Preeclampsia	Abnormal fetal position
Neonatal features and follow-up outcomes							
Infant No	1a	1b	2a	2b	3	4	5
CMV DNA in amniotic fluid (copies/mL)	4.18 × 10^4^	1.19 × 10^4^	7.88 × 10^6^	2.13 × 10^4^	5.81 × 10^7^	1.34 × 10^3^	2.54 × 10^2^
CMV IgG in cord blood	positive	positive	positive	positive	positive	Not available	Not available
CMV IgM in cord blood	positive	positive	positive	negative	negative	Not available	Not available
Prenatal ultrasound	Normal	IUGR at GW 25^+1^, abnormal umbilical blood flow at GW 29^+5^	Normal	Normal	Normal	Normal	Normal
Apgar score (1/5 min)	8/9	8/9	9/10	9/10	9/10	9/10	9/10
Birth weight (g)	2180	1390	3040	2920	4175	2980	3300
Birth length (cm)	44	39	50	48	52	50	50
Clinical features after birth *	Neonatal pneumonia and respiratory distress syndrome, necrotizing enteritis	IUGR, neonatal pneumonia and respiratory distress syndrome, necrotizing enteritis, ileum perforation	No	No	No	Neonatal hyperbilirubinemia	No
Children follow-up (months) ^†^	Normal (56)	Normal (56)	Normal (54)	Normal (54)	Normal (49)	Normal (35)	Normal (18)

* None of the infants showed CMV-related signs such as petechiae, tachypnea, hepatomegaly, splenomegaly, thrombocytopenia, seizures, microcephaly, or hypotonia at birth. ^†^ All seven children had normal vision, hearing, and mental development and had no neurological sequelae at the follow-up. IUGR: intrauterine growth restriction.

## Data Availability

The data are available upon reasonable request.

## Supplementary Materials

The following supporting information can be downloaded at https://www.mdpi.com/article/10.3390/pathogens14090894/s1: Table S1: Indications for Cesarean section in 695 pregnant women.

## Abbreviations

The following abbreviations are used in this manuscript:

CMV	Cytomegalovirus
qPCR	quantitative real-time polymerase chain reaction
AI	Avidity index
SD	Standard deviation
