# Supplementary material for: Transplacental Transmission of Cytomegalovirus (CMV) in Pregnant Women with Positive Anti-CMV IgG and Negative Anti-CMV IgM in Highly CMV Seropositive Region
Source: Pathogens. 2025 Sep 5;14(9):894. doi: 10.3390/pathogens14090894 (PMC12472506; doi:10.3390/pathogens14090894)
Supplement: Supplementary file 1 [file pathogens-14-00894-s001.zip › pathogens-3850102-supplementary.pdf]

Table S1 Indications for Cesarean section in 695 pregnant women*

Indications	Number	Proportion (%)
Scarred uterus	334	48.1
Twin pregnancy	137	19.7
Fetal distress	42	6.0
Abnormal labor process	40	5.8
Macrosomia	32	4.6
Abnormal fetal position	28	4.0
Gestational diabetes	20	2.9
Preeclampsia	17	2.4
Precious baby	12	1.7
Oligohydramnios	11	1.6
Placenta previa	8	1.2
Uterine fibroids	4	0.6
Placental abruption	3	0.4
Intrahepatic cholestasis	3	0.4
Cardiac insufficiency	2	0.3
Abnormal umbilical blood flow	1	0.1
Maternal scoliosis	1	0.1
Total	695	100

*If a pregnant woman had multiple indications for Cesarean section, only the main indication was included.
